# Activated platinum in gallium-based room-temperature liquid metals for enhanced reduction reactions†

**DOI:** 10.1039/d3ra06571e

**Published:** 2023-10-16

**Authors:** Nichayanan Manyuan, Hideya Kawasaki

**Affiliations:** a Department of Chemistry and Materials Engineering, Kansai University 3-3-35, Yamate-cho, Suita Osaka 564-8680 Japan hkawa@kansai-u.ac.jp

## Abstract

Room-temperature gallium-based liquid metals (LMs) have recently attracted significant attention worldwide for application in catalysis because of their unique combination of fluidic and catalytic properties. Platinum loading in LMs is expected to enhance the catalytic performance of various reaction systems. However, Pt-loaded methods for Ga-based LMs have not yet been sufficiently developed to improve the catalytic performance and Pt utilization efficiency. In this study, a novel method for the fabrication of Pt-incorporated LMs using Pt sputter deposition (Pt(dep)-LMs) was developed. The Pt(dep)-LMs contained well-dispersed Pt flakes with diameters of 0.89 ± 0.6 μm. The catalytic activity of the Pt(dep)-LM with a Pt loading of ∼0.7 wt% was investigated using model reactions such as methylene blue (MB) reduction and hydrogen production in an acidic aqueous solution. The Pt(dep)-LMs showed a higher MB reduction rate (three times) and hydrogen production (three times) than the LM loaded with conventional Pt black (∼0.7 wt%). In contrast to the Pt(dep)-LMs, solid-based Ga with a Pt loading of ∼0.7 wt% did not catalyze the reactions. These results demonstrate that Pt activation occurred in the Pt(dep)-LMs fabricated by Pt sputtering, and that the fluidic properties of the LMs enhanced the catalytic reduction reactions. Thus, these findings highlight the superior performance of the Pt deposition method and the advantages of using Pt-LM-based catalysts.

## Abstract 2

Pt-incorporated liquid metal using Pt sputter deposition was developed, showing high catalytic activity on methylene blue reduction and hydrogen production in an acidic aqueous solution.

## Graphical abstract

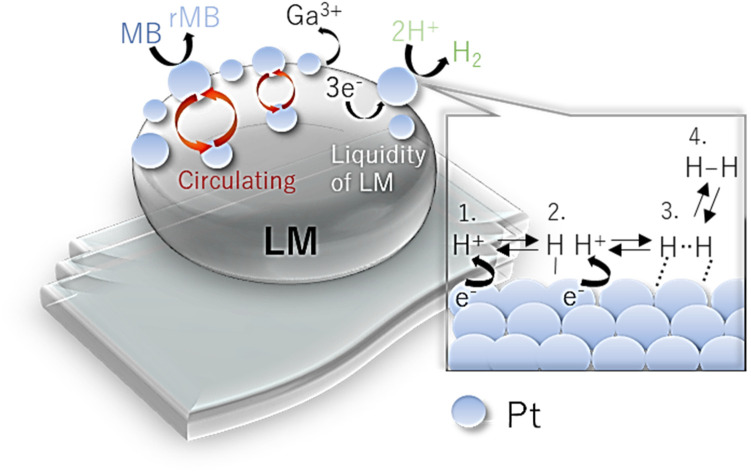

## Introduction

Room-temperature liquid metals (LMs) are a group of low-melting-point alloys. One well-known example is Ga-based eutectic alloys such as eutectic gallium-indium (EGaIn), which offer several advantages over other LMs such as Hg.^1,2^ Ga-based LMs exhibit low toxicity and low vapor pressure, and are thus easily tailored. The high electrical and heat conductivities of Ga-based LMs have led to their applications in heat transfer and cooling systems, electronic components, soft robotics, microfluidics, and surface coatings.^3–8^ In addition, Ga-based LMs are effective catalysts for various chemical reactions, including hydrogenation, dehydrogenation, oxidation, and reduction.^9–14^ The concept of supported catalytically active liquid metal solutions (SCALMSs) has recently emerged as a novel approach in LM catalysis.^15^ In SCALMS, a low-melting metal such as Ga, which is typically catalytically inactive, is doped with a small amount of a catalytically active metal such as Pd, Rh, Ni, or Pt.^15–19^

Platinum has been extensively applied as a catalyst in various chemical reactions, providing significant advantages of promoting specific reactions and improving overall efficiency.^20^ The excellent catalytic properties of Pt are invaluable in LM catalysis, because it enhances chemical processes in various industries, such as fuel production, chemical synthesis, and hydrogen generation.^16,19,21^ For GaPt-SCALMS catalysts, Raman *et al.* demonstrated the high catalytic GaPt-SCALMS catalysts supported on different materials such as silica (SiO_2_), alumina (Al_2_O_3_), and silicon carbide (SiC).^16^ Oloye *et al.* reported an electrocatalytically active GaPt-SCALMS catalyst for the hydrogen evolution reaction (HER).^22^ Kumar *et al.* demonstrated the excellent efficiency of the electrochemical oxidation process such as the methanol oxidation reaction (MOR) and oxygen reduction reaction (ORR), surpassing the performance of a commercial Pt/C catalyst.^23^ Furthermore, Zhang *et al.* reported that GaPt nanoparticles provided up to 98% propylene selectivity at 600 °C.^24^ The metals can form ordered intermetallic compounds (*e.g.* Ga_37_Pt and Pd_2_Ga) as catalytic active components.^25–27^ The scientific interest in this new frontier of heterogeneous catalysis is evidenced by the recent explosion of research on supported single metal atom catalysis.^28–32^ The precious metal is fully dissolved in the liquid Ga matrix during the reaction process and showcases remarkable mobility, causing the creation of catalytic sites consisting of single atoms at the liquid alloy–gas interface. This specific atomic behaviour has been verified for GaPt, with a minimal Pt loading (<0.1 × 10^−3^ at%), through the electrochemical oxidation of methanol by Rahim *et al.*^12^

Galvanic displacement from Pt salts has been used to fabricate GaPt-SCALMS catalysts.^22,23,33–35^ This process involves the immersion of a Ga-based LM in a solution containing Pt ions, such as K_2_PtCl_4_. Ga acts as a reducing agent and donates electrons to the Pt ions, forming a Pt coating on the liquid metal surface. Factors such as the reaction kinetics, temperature, and metal ion concentration can affect the deposition rate and uniformity. The galvanic displacement process requires the systematic optimization of various parameters to control the thickness and morphology of the Pt deposits. The precise control of Pt deposition by galvanic displacement on LMs is challenging.

Sputter deposition is a relatively simple and convenient method for depositing Pt on LMs. This method is a form of physical vapor deposition (PVD) that involves bombarding a target material with high-energy particles,^36–38^ causing metal atoms or clusters to be ejected from the target and deposited onto the substrate, resulting in the formation of nanoparticles or a thin film.^39,40^ Sputter deposition has several advantages over other deposition techniques, such as the precise control of Pt loaded amount by adjusting the deposition time and rate. Furthermore, sputter deposition can produce high-purity Pt because the process occurs in vacuum, thereby minimizing the risk of contamination from atmospheric gases. This is particularly important for applications requiring pure Pt for catalysis.^36,37^ Additionally, sputter deposition can be performed at relatively low temperatures, thereby reducing the risk of substrate damage or deformation. This is valuable to ensure the structural integrity of delicate LM substrates. However, the use of Pt sputter deposition on LMs to fabricate GaPt-SCALMSs has not been reported thus far.

In this study, a novel and facile method for fabricating GaPt-SCALMS by Pt sputtering deposition on an EGaIn LM is reported. Two methods are used: (i) Pt sputter deposition, denoted as Pt(dep)-LM, and (ii) Pt black dispersion achieved by simple mixing, denoted as Pt(black)-LM. The catalytic activities of the two GaPt-SCALMSs are evaluated using two model reduction reactions: methylene blue (MB) reduction by monitoring the ultraviolet-visible (UV-vis) spectra and hydrogen production in an acidic aqueous solution. The Pt sputter deposition approach enabled good dispersion of Pt particles in Pt(dep)-LM, compared to the aggregation of Pt black in Pt(black)-LM. Pt(dep)-LM exhibited considerably higher MB reduction and hydrogen production rates than Pt(black)-LM. The accelerated reaction activity of Pt(dep)-LM is discussed based on the well-dispersed Pt particles in the LM. Finally, this study demonstrates that the fluidity of Pt(dep)-LM contributes to enhanced reduction reactions *via* the dynamic movement of Pt particles.

## Experimental section

### Materials and chemicals

Ga and In with 99.99% purity were purchased from Kojundo Chemical Lab. Co., Ltd, Japan. Pt black powder (≤20 μm, ≥ 99.95% purity) and hydrochloric acid (HCl) solution were purchased from Sigma-Aldrich. MB with 98.5% purity was purchased from Wako Pure Chemical Industries, Ltd, Japan.

### Preparation of Pt-liquid metal and Pt-solid metal

#### Preparation of the liquid metal

The LM was prepared by mixing 75.5 wt% Ga (1.54 g) and 24.5 wt% In (0.5 g). The mixture was placed in a vessel and heated on a hot plate at 180 °C to produce the EGaIn LM.

#### Preparation of Pt(dep)-LM

**Fig. 1:**
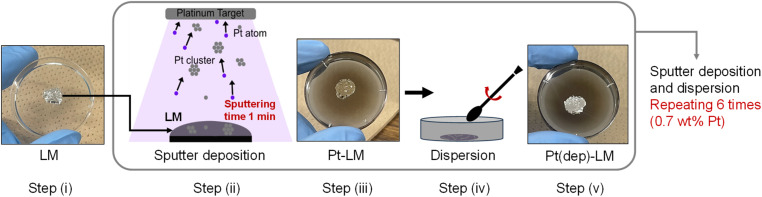
Schematic illustration of the fabrication of Pt(dep)-LM *via* sputter deposition.

The as-fabricated LM (0.05 g) was placed in a Petri dish and then Pt was deposited onto the LM by magnetron sputter deposition (MSP-1S Vacuum Device Co., Ltd, Japan) for 1 min at room temperature (23 °C), followed by hand mixing with a spatula for dispersion. The Pt sputter deposition and dispersion process were repeated six times, obtaining Pt-deposited LMs with a Pt loading of 0.70 wt%, as shown in Fig. 1. To determine the optimal loading of Pt required for the best catalytic performance, Pt(dep)-LMs with different Pt loadings of 0.1, 0.3, 0.7, and 1.0 wt% Pt were prepared using different Pt sputtering times of 1, 3, 6, and 9 min, respectively. The amount of Pt loading was evaluated based on the relationship between the Pt deposition amount and the deposition time (Fig. S1†). The Pt loading (estimated from a calibration curve) in the Pt(dep)-LMs was confirmed using X-ray fluorescence (XRF; JSX-1000S, JEOL, Japan).

#### Preparation of solid Pt-gallium (Pt(dep)-SM)

For Pt(dep)-SM, pure Ga with a melting point of 29.76 °C was used as a solid-state catalyst at 23 °C. Ga powder (0.05 g) was melted at 180 °C, and then Pt was deposited onto the molten Ga by Pt sputter deposition for 1 min. The Pt-deposited Ga was heated on a hot plate at 80 °C to ensure complete melting of the Ga and mixed with a spatula. The sputter deposition and mixing steps were repeated six times under the same conditions as those for Pt(dep)-LM. Subsequently, the as-fabricated Pt(dep)-SM was placed in a freezer at −20 °C overnight to ensure solidification. Thus, solid Pt(dep)-SM (0.7 wt% Pt loading) was obtained.

#### Preparation of Pt-LM by Pt black mixing (Pt(black)-LM)

For comparison with Pt sputter deposition, Pt black powder (0.35 mg) was mixed with the LM (0.05 g) using a spatula, as shown in Fig. S2.† Thus, Pt(black)-LM (0.7 wt% Pt loading) was obtained.

### Model reactions on activated Pt-LM

#### MB reduction by Pt-LM

Firstly, MB (1.0 mg) was added to 0.1 M HCl (100 mL) in a 150 mL vial to prepare a MB stock solution. Subsequently, the prepared MB solution (20 mL) and Pt(dep)-LMs (30 mg) were mixed in 100 mL vials, and the mixtures were shaken using a shaking machine (MMS-1020 Tokyo Rikakikai Co., Ltd, Japan) at 100 rpm for 100 min. The absorbance of the aqueous MB solution at 665 nm was monitored every 20 min using a UV-vis spectrophotometer (V-670, JASCO Corporation, Tokyo, Japan).

For MB reduction using LM (no Pt), Pt(black)-LM, and Pt(dep)-SM, we utilized the aforementioned procedure. To examine the effect of HCl concentration on MB reduction, we changed the HCl concentration using the above method. The reduction fraction of the dye (*R*_MB_) can be expressed in the following equation using eqn (1).1
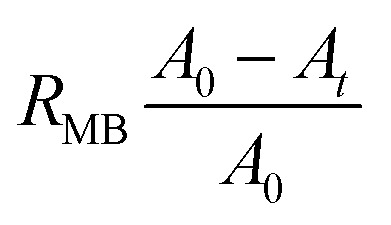
where *A*_0_ and *A*_*t*_ are the MB absorbance values at the onset of the reaction and at time *t* (min), respectively. The reduction of MB dye exhibits pseudo-first-order kinetics relative to the reaction time, as reported in the literature.^41–43^

The rate constant is determined as follows:

2 −ln(*C*_*t*_/*C*_0_) = −ln(*A*_*t*_/*A*_0_) = *k*_app_ × *t*

where *C*_0_, *C*_*t*_, and *k*_app_ (min^−1^) are the initial MB concentration, the MB concentration at different time intervals, and the apparent reaction rate constant, respectively.

#### Hydrogen production by Pt-LM

The MB aqueous solution (20 mL) prepared with 0.1 M HCl was added to the Pt(dep)-LMs (30 mg) in 100 mL vials, and the mixtures were shaken at 100 rpm for 100 min. The amount of hydrogen gas (ppm) in a 100 mL vial produced from the MB aqueous solution in the presence or absence of the Pt(dep)-LMs was measured using a hydrogen gas sensor (Hydrogen Meter UPX4, UP. Co., Ltd, Japan) every minute. The same procedure was used for hydrogen production by the Pt(black)-LM and Pt(dep)-SM.

### Characterization of Pt-LMs

Transmission electron microscopy (TEM; JEM-1400F, JEOL, Tokyo, Japan) was performed at an acceleration voltage of 100 kV to characterize the morphology of Pt(dep)-LM and Pt(black)-LM supported on TEM grids. Field emission-scanning electron microscopy (FE-SEM; JSM-6700, JEOL, Japan) with an acceleration voltage of 15–25 kV was used to observe the Pt particles in the Pt-LMs. Scanning electron microscopy coupled with energy-dispersive X-ray spectroscopy (SEM-EDS; JCM-6000Plus NeoScope, JEOL, Japan) mapping was performed to determine the Pt distribution on the surface of the Pt-LMs. X-ray photoelectron spectroscopy (XPS) with a monochromatic Al Kα X-ray source (PHI Quantera II XPS, Physical Electronics, Inc., USA) was used to characterize the binding energies of the Pt-LMs. X-ray diffraction (XRD) patterns of the Pt(dep)-LMs were obtained using a D2 PHASER XRD diffractometer (Bruker, D2 PHASER, Germany).

## Results and discussion

### MB reduction by Pt(dep)-LM

**Fig. 2:**
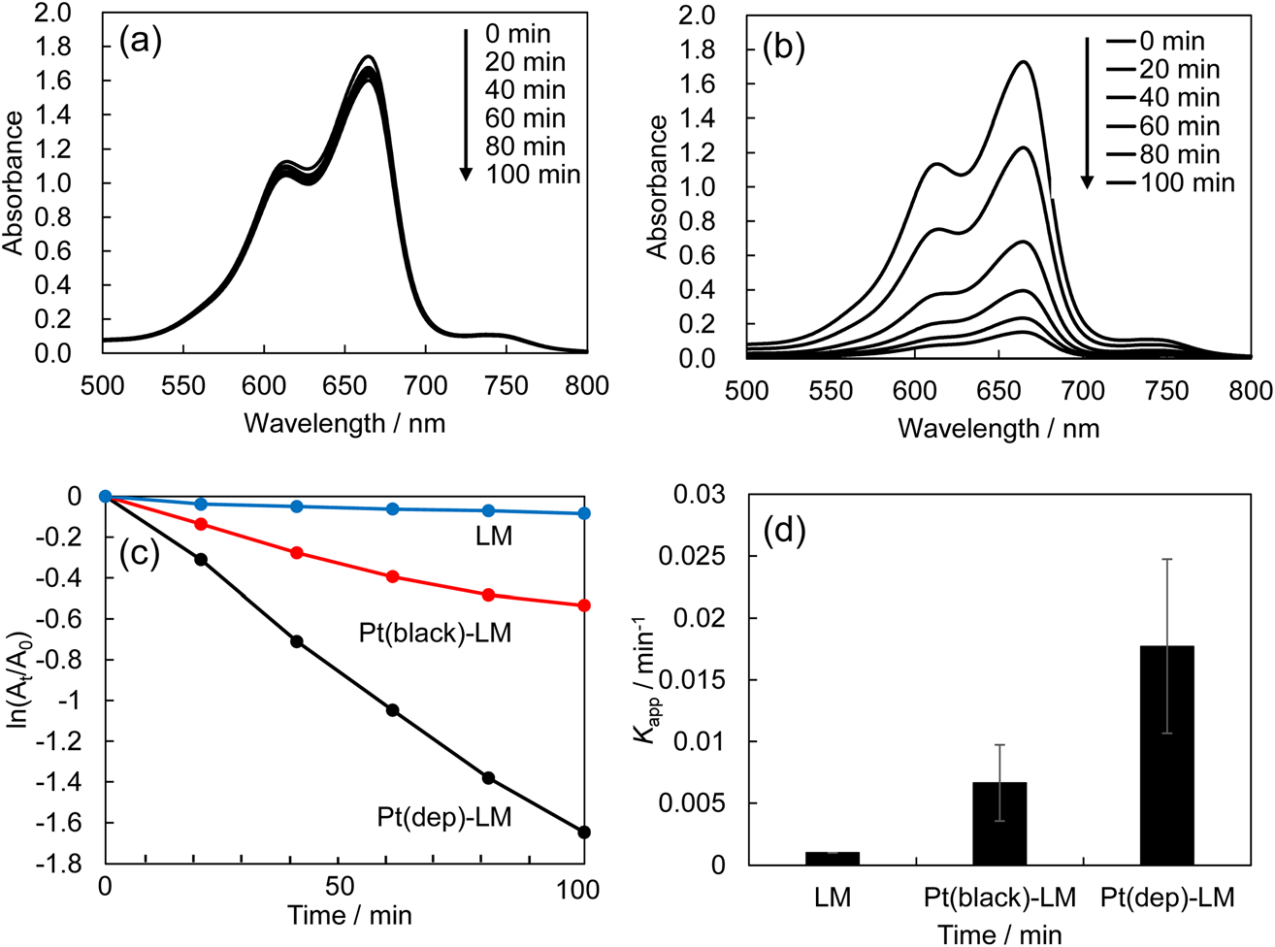
Time-dependent UV-Vis spectra of the reduction of MB in an acidic aqueous solution (0.1 M HCl) in the presence of (a) LM (no Pt) and (b) Pt(dep)-LM, (c) and (d) comparison of the different methods on MB reduction.

Metallic nanoparticles are well known for their catalytic activity in electron charge-transfer reactions.^44^ MB reduction by the Pt(dep)-LMs was investigated to evaluate the applicability of the Pt(dep)-LMs. This reaction is known to proceed *via* a pathway that involves the decolorized formation of leuco-MB species, which can be monitored based on the decrease in absorbance at approximately 665 nm.^45^Fig. 2 shows the MB reduction of MB in an acidic aqueous solution (0.1 M HCl) containing LM (without Pt) or the Pt(dep)-LMs. The UV-vis spectra of LM exhibited almost no change in MB absorbance (*i.e.*, no decolorized MB) (Fig. 2a). In contrast, a distinct decrease in absorbance was observed for the Pt(dep)-LMs (Fig. 2b), indicating the higher catalytic activity of Pt(dep)-LM for MB reduction, as confirmed by the faster MB reduction for Pt(dep)-LM (*k*_app_ = 0.0168 min^−1^) than for LM (*k*_app_ = 0.0008 min^−1^) (Fig. 2c and d).

The electron transfer from Pt to MB is facilitated by the Pt(dep)-LMs because the liberated electrons from the Ga oxidation process (Ga → Ga^3+^ + 3e^−^) can be injected into the Pt deposited on the LM surface, resulting in the faster MB reduction compared to LM without Pt. We recorded the *k*_app_ reaction rate during the initial reaction period of 60 min for MB reduction and the first 10 min for hydrogen production (described later). During these stages, the compositional changes were relatively small: the Ga ratio in Pt (dep)-LM decreased by approximately 3% after a reaction period of 1 h. We propose that assessing the activity of the Pt-LMs by comparing the initial reaction rates is a valid approach.

The effect of the amount of Pt deposited in the Pt(dep)-LM on the MB reduction was further investigated. Among the investigated Pt contents, which ranged from 0.1 to 1.0 wt% Pt, the Pt(dep)-LM with a 0.7 wt% Pt loading exhibited the largest value of *R*_MB_ after 100 min reaction (Fig. S3a†) and the largest MB reduction rate (Fig. S3b†). Considering the 0.1–0.3 wt% Pt loadings, the Pt content on the Pt(dep)-LM surface was relatively small. In contrast, a high Pt loading of 1.0 wt% caused Pt aggregation or the size growth in the LM, reducing its MB reduction. Therefore, the 0.7 wt% Pt loading was selected as the optimal Pt deposition condition for Pt(dep)-LM catalysis. Notably, the MB reduction rate per Pt mass loading (*K*_app_/min^−1^ mg^−1^) was highest at a 0.1 wt% Pt loading (Fig. S4†).

It is well known that metallic Ga undergoes self-passivation by forming an ultrathin Ga oxide layer upon air exposure.^46,47^ The Ga oxide can be partially removed in acid solutions with concentrations higher than 10^−3^ HCl mol L^−1^; the removal degree depends on the HCl concentration.^46^ Ga oxide on the LM surface can inhibit the MB reduction reaction over Pt(dep)-LM. Therefore, the effect of HCl concentration on the MB reduction rate over Pt(dep)-LM was evaluated (Fig. S5†).

Pt(dep)-LM exhibited a higher MB reduction (*k*_app_ = 0.0168 min^−1^) in 0.1 M HCl than in 0.01 M HCl (*k*_app_ = 0.0006 min^−1^)and water (*k*_app_ = 0.0003 min^−1^). The Ga oxide on the LM surface may have hindered electron transfer from Ga to Pt during the MB reduction reaction.

### MB reduction by Pt(black)-LM

Pt black powder has a unique structure with a high surface area, typically consisting of porous nanoparticle aggregates. The increased surface area provides more Pt sites for catalytic reactions, enhancing overall catalytic activity and efficiency.^48–50^ The TEM images of Pt black show porous nanoparticle aggregates with a primary particle size of <10 nm (Fig. S6†). In addition, the performance of Pt(black)-LM in the MB reduction reaction was compared with that of Pt(dep)-LM (Fig. 2c and d). Despite having the same Pt loading (∼0.7 wt%), Pt(black)-LM exhibited a lower MB reduction (*k*_app_ = 0.0055 min^−1^) than that of Pt(dep)-LM (*k*_app_ = 0.0168 min^−1^), clearly indicating that it is less active than Pt(dep)-LM. This result suggests that Pt deposited on the LM by the sputter deposition method (*i.e.*, Pt(dep)-LM) provided abundant catalytic Pt sites on the LM surface for MB reduction, resulting in a higher MB reduction rate compared to Pt(black)-LM. In the MB reduction reaction, the Ga in Pt-LM was oxidized. Therefore, Ga oxidation is expected to be more dominant in the Pt (dep)-LMs than in the Pt (black)-LMs. A more substantial decrease was observed in Pt (dep)-LM (∼3%) than in Pt (black)-LM (∼0.4%) after 60 min of the MB reduction reaction using SEM-EDS analysis.

### Pt flake-dispersion in Pt(dep)-LM

**Fig. 3:**
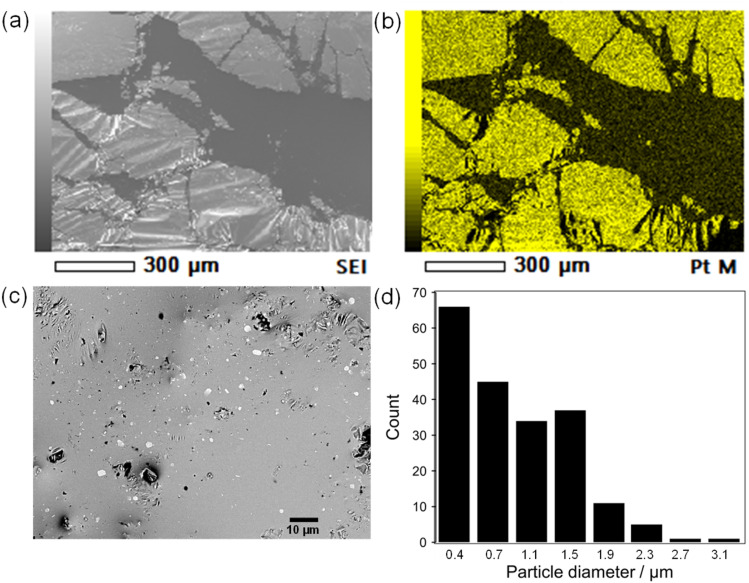
(a) and (b) show SEM images of Pt(dep)-LM before mixing (1 min of deposition), (c) FE-SEM image of Pt(dep)-LM after mixing, and (d) size distribution of Pt(dep)-LM.

The Pt sputter deposition (1 min) and mixing steps were repeated six times to prepare Pt(dep)-LM with highly dispersed. The SEM image of Pt(dep)-LM (Fig. 3a) was obtained to observe the Pt particles on the LM surface before hand mixing, (Fig. 1, step (iii)), showing a Pt film on the LM surface, which is supported by the SEM-EDS analysis results (Fig. 3b). XRD analysis of the Pt film on the LM surface revealed a small Pt crystal size of approximately 8–10 nm (Fig. S7†).

**Fig. 4:**
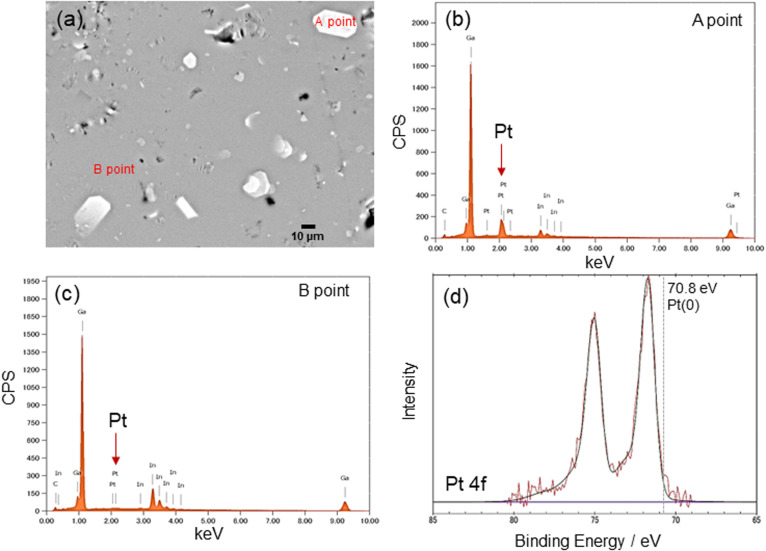
(a) FE-SEM image, (b) and (c) EDS point analysis, and (d) Pt 4f XPS spectra of Pt(dep)-LM.

In addition, Pt sputtering was performed on a TEM grid to investigate the morphology of the sputter-deposited Pt. The morphology of Pt in Pt(dep)-LM could not be directly characterized by TEM. Thus, a TEM grid was used as the substrate to observe the sputter-deposited Pt (Fig. S8†). The TEM image shows a Pt nanoisland with a crack-like boundary, which is consistent with the morphology of a Pt film prepared by the sputtering method.^51^ Ga-based LM penetrates the crack-like boundary of metals owing to its good wettability with various metals, which is known as a liquid-metal-embrittlement crack.^52^ Thus, the crack-like boundary in the Pt film may have initiated fragmentation by hand mixing (Fig. 1, step (iv)) into well-dispersed flake-like Pt particles on the LM surface, as shown in Fig. 3c and S9.† In addition, the fluidity allows the Pt films to be easily dispersed into the Pt particles, thereby increasing the surface area of the catalytic Pt sites. The particle size distribution of Pt(dep)-LM has an average diameter of 0.89 ± 0.6 μm (Fig. 3d). The flake-like particles in Pt (dep)-LM were confirmed to be Pt by SEM-EDS point analysis (Fig. 4a). The Pt signals for the flake-like particles were observed at point A in Fig. 4b, but fewer Pt signals were detected in the area without flake-like particles, as shown at point B in Fig. 4c. Thus, the Pt particles in Pt(dep)-LM were uniformly dispersed on the LM surface, leading to a high MB reduction ability. As a result, Pt(dep)-LM exhibits dispersed Pt particles throughout the SEM images, while Pt(black)-LM displays unevenly distributed Pt particles that form aggregates (as shown in Fig. S10† SEM image). Consequently, this results in a decrease in the number of exposed Pt particles on the LM surface and lower MB reduction performance.

The XPS spectrum of Pt(dep)-LM was measured to evaluate the electronic state of Pt. Fig. 4d shows the Pt 4f XPS spectrum of Pt(dep)-LM, in which the Pt 4f7/2 peak was observed at 71.8 eV. Thus, the binding energy of the Pt 4f7/2 peak of Pt(dep)-LM was higher than that of pure metallic Pt (70.8 eV).^23^ The decreased electron density around the surface of Pt can enhance its electron-accepting ability, thereby enhancing the high catalytic MB reduction performance of Pt(dep)-LM.

### Hydrogen production by liquid-based Pt(dep)-LM

**Fig. 5:**
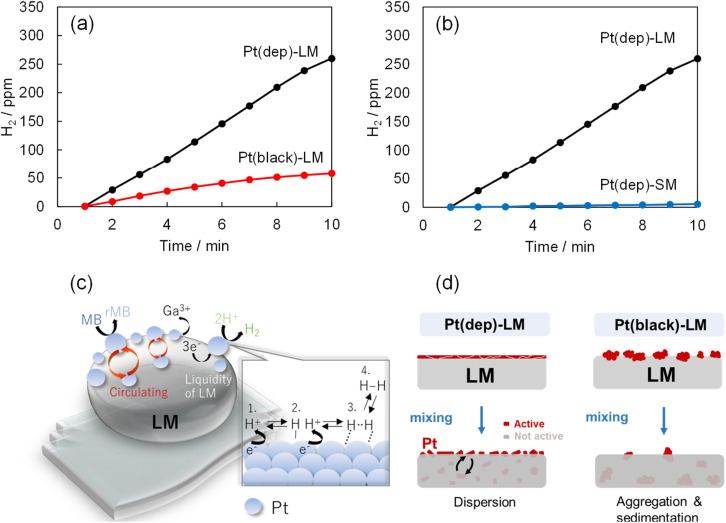
Comparison of the hydrogen production rate of (a) Pt(dep) and Pt(black)-LM and (b) Pt(dep)-LM and Pt(dep)-SM. (c) and (d) schematic of the proposed reaction mechanism over Pt on the LM surface. In (b), the hydrogen generation start time is normalized to start at 1 min due to the long induction time (∼100 min) for Pt(dep)-SM.

Pt nanoparticles have been extensively used in hydrogen production research and industrial applications.^53^ The combination of Pt and the unique properties of LMs (*i.e.*, Pt-LM) can enhance hydrogen production. In this study, hydrogen gas bubbles formed on the Pt(dep)-LM surface in an 0.1 M HCl/MB aqueous solution owing to a side reaction of MB reduction (as shown in Movie S1†). In contrast, hydrogen gas bubbles were not generated by LM (no Pt). Ga in Pt(dep)-LM was dissolved in 0.1 M HCl, forming Ga^3+^ and 3e^−^. In aqueous solution, protons (H^+^) are present, which are likely adsorbed by the Pt particles on the LM surface to form Pt–H species according to the general Pt activation mechanism for hydrogen production.^54^ These adsorbed hydrogen atoms combine to form hydrogen molecules (H_2_), which desorb from the Pt catalyst surface, releasing H_2_ gas (as shown in a schematic Fig. 5c). In addition, a control experiment on hydrogen production by the Pt(dep)-LM without MB was conducted (Fig. S11†). The hydrogen reaction rate in the absence of MB (43.7 ppm min^−1^) increased compared with that in the presence of MB (28.1 ppm min^−1^). This corresponds with our hypothesis that the electrons generated by Ga oxidation are utilized for both MB reduction and hydrogen production(as shown in a schematic Fig. 5c).

The hydrogen production rates of Pt(dep)-LM and Pt(black)-LM in a 0.1 M HCl/MB aqueous solution were compared, as shown in Fig. 5a. The hydrogen production rate of Pt(black)-LM (8.6 ppm min^−1^) was considerably slower than Pt(dep)-LM; thus, the faster hydrogen production rate indicates the presence of more Pt sites on the Pt(dep)-LM surface than on the Pt(black)-LM surface. Moreover, the liquidity of the LM enables the smaller Pt particles on the Pt(dep)-LM surface to flow inward and outward, leading to the continuous regeneration of active Pt particles, which prevents catalyst deactivation and maintains a high catalytic performance over prolonged periods. However, for Pt(black)-LM, the aggregation of Pt black due to its poor dispersibility in the LM provided less Pt on the LM surface compared to Pt(dep)-LM (as schematically shown in Fig. 5d).

### Hydrogen production by solid-based Pt(dep)-SM

To investigate the effect of LM liquidity on hydrogen production in the liquid-based Pt(dep)-LM, hydrogen production by the solid-based Pt(dep)-SM was evaluated at 23 °C. Pt(dep)-SM exhibited a considerably slower hydrogen production rate (0.64 ppm min^−1^) than Pt(dep)-LM (Fig. 5b). A similar trend was observed for the MB reduction rate of Pt(dep)-SM, which was slower than that of Pt(dep)-LM (Fig. S12†). The liquid substrate of Pt(dep)-LM likely possesses better diffusion and mobility for the Pt particles than the solid substrate of Pt(dep)-SM. The fluidic nature of the LM facilitates the transport of Pt particles to the LM surface. The increased mobility allowed the Pt sites to dynamically reposition themselves within the liquid, enhancing their ability to interact with the reactants (H^+^), and thus improving the hydrogen production rate. In the solid substrate of Pt(dep)-SM, Pt mobility is limited and the substrate typically has a fixed surface. Consequently, the contact of Pt(dep)-SM with the reactant is reduced, and thus it is more prone to the deactivation of hydrogen production.

The induction time of Pt catalysis refers to the initial period, during which little or no hydrogen evolution occurs, and is followed by a rapid increase in the hydrogen generation rate.^55,56^ Notably, an induction time was observed during hydrogen production by Pt(dep)-LM and Pt(dep)-SM. The induction time for Pt(dep)-SM (∼100 min) was significantly longer than that for Pt(dep)-LM (1∼5 min). The shorter induction time of Pt(dep)-LM is advantageous because the Pt in Pt(dep)-LM can rapidly reach its active state and generate hydrogen, leading to faster and more efficient hydrogen production. The origin of the long induction time for Pt(dep)-SM is unclear, and different factors may contribute to this phenomenon. The induction time may be related to the time required for the catalyst surface to restructure and reach an active state suitable for hydrogen evolution. This restructuring may involve the formation of the removal of Ga oxide, which inhibits the reaction. Research is underway to further understand and control the induction time to optimize Pt catalysis in LMs for efficient hydrogen production in the future.

Notably, there are limitations to the fabrication of the Pt-LMs under the reaction conditions used in this study. Ga, a crucial component of the Pt-LM catalysts, is continuously consumed as the reactions proceed, and thus leads to a decrease in the catalytic capacity. Consequently, the formation of a solid material with a low Ga content (∼37%) was observed after a one-day reaction period, coupled with a decrease in catalytic activity. As part of our future research endeavors, however, our proposed methods to activate Pt-LMs *in situ* will be helpful for reaction systems with different reducing agents, in addition to those involving electrons provided by Ga oxidation. This approach aims to address the issue of catalyst durability and expand the potential application scope of Pt-LMs in the future.

## Conclusion

This study demonstrated a novel fabrication method for GaPt SCALMs using the Pt sputter deposition method to achieve high MB reduction and hydrogen production rates. The Pt sputter deposition method produced well-dispersed and catalytically active flake-like Pt particles in Pt(dep)-LM. These characteristics are crucial for enhancing the efficiency of the reduction reactions in Pt(dep)-LM. Pt(black)-LM contained aggregated Pt particles in the LM (*i.e.*, poor Pt dispersion) and less Pt on the LM surface compared with Pt(dep)-LM, decreasing the MB reduction and hydrogen production rates.

The liquid nature of the Pt(dep)-LM provides abundant Pt sites on the LM surface for MB reduction and hydrogen production, compared with the solid-based Pt(dep)-SM, which may be due to the continuous regeneration of Pt particles from the interior of LMs.

Moreover, Pt(dep)-LM exhibited a shorter induction time than Pt(dep)-SM, which allowed for faster activation and more efficient hydrogen production. Therefore, this study demonstrates that Pt activation occurs by Pt sputter deposition on LMs, and that the fluidic properties of the LMs enhance catalytic reactions such as MB reduction and hydrogen production.

A vapor deposition process, including sputter deposition, would fabricate various metals or metal oxides with high purity upon the LM substrate, where they nucleate and form catalytic nanoparticles. Thus, this study highlights the superior performance of the vapor deposition method for fabricating high-performance LM-based catalysts.

## Author contributions

NM and HK conceived the idea of the study. NM conducted experiments and contributed to the interpretation of the results. NM drafted the original manuscript. HK supervised the conduct of this study. All authors reviewed the manuscript draft and revised it critically on intellectual content. All authors approved the final version of the manuscript to be published.

## Conflicts of interest

There are no conflicts to declare.

## Supplementary Material

RA-013-D3RA06571E-s001

RA-013-D3RA06571E-s002
