# Supplementary material for: Activated platinum in gallium-based room-temperature liquid metals for enhanced reduction reactions
Source: RSC Adv. 2023 Oct 16;13(43):30273–80. doi: 10.1039/d3ra06571e (PMC10577643; doi:10.1039/d3ra06571e)
Supplement: RA-013-D3RA06571E-s002 [file RA-013-D3RA06571E-s002.pdf]

Electronic Supplementary Material (ESI) for RSC Advances.
This journal is © The Royal Society of Chemistry 2023

Supporting Information

Activated platinum in gallium-based room-temperature liquid metals for enhanced reduction reaction

Nichayanan Manyuan and Hideya Kawasaki*

Department of Chemistry and Materials Engineering, Kansai University, 3-3-35, Yamate-cho, Suita, Osaka 564-8680, Japan.

*Corresponding author: E-mail: hkawa@kansai-u.ac.jp

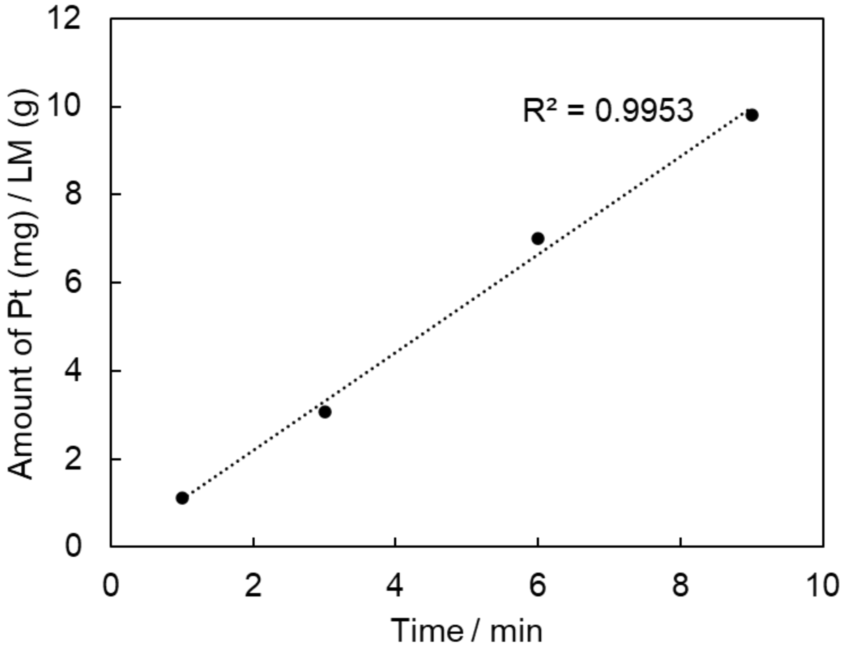

Figure S1 Calibration curve of the Pt loading at different sputtered time.

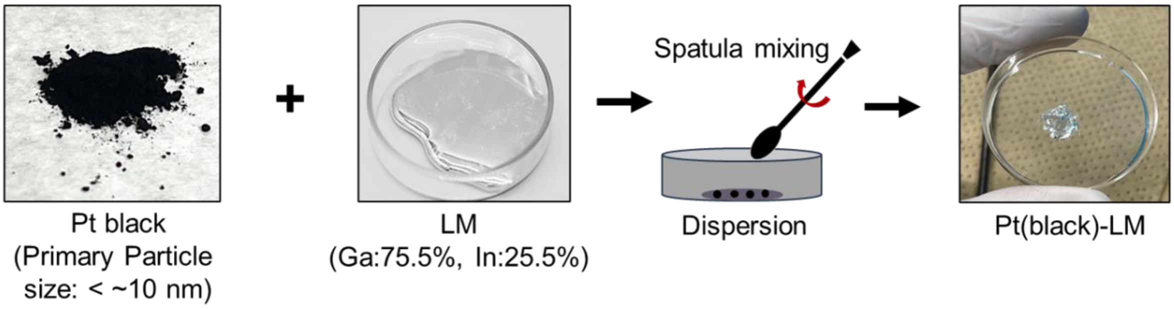

Figure S2 Schematic illustration of the fabrication of Pt(black)-LM via Pt black mixing.

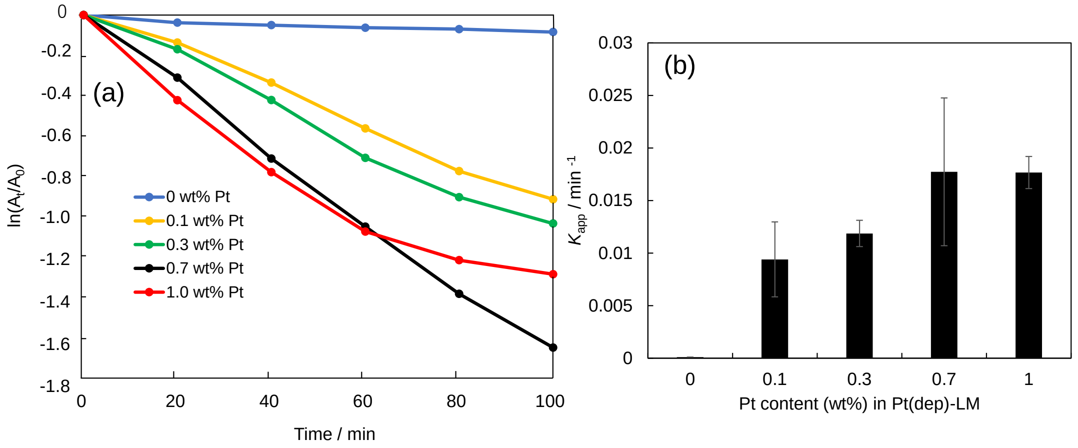

Figure S3 (a) Time-dependent reduction of MB in an acidic aqueous solution (0.1 M HCl) in the presence of Pt(dep)-LM with the different Pt content (wt %) and (b)comparison of the different Pt content (wt %) in Pt(dep)-LM on MB reduction rate.

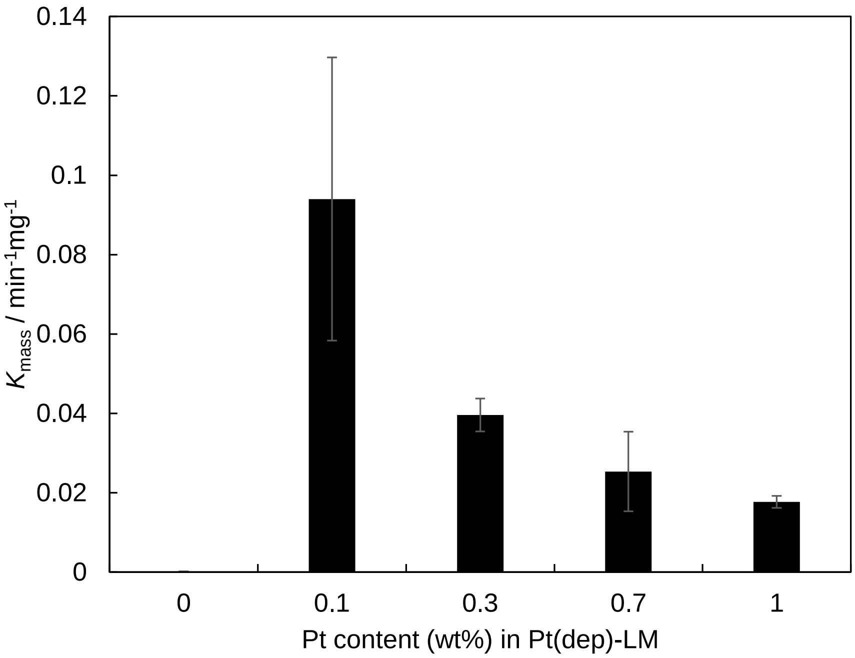

Figure S4 Comparison of the different Pt content in Pt(dep)-LM on MB reduction rate per Pt mass (K_{mass}).

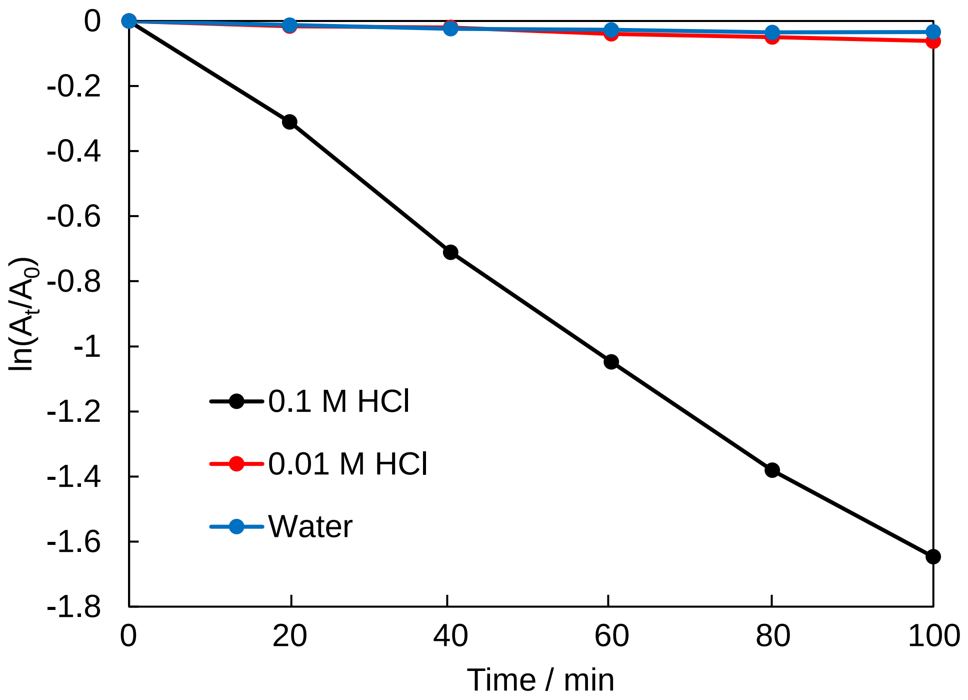

Figure S5 Comparison of MB reduction rate in different HCl solution including 0.1M HCl, 0.01 HCl, and water.

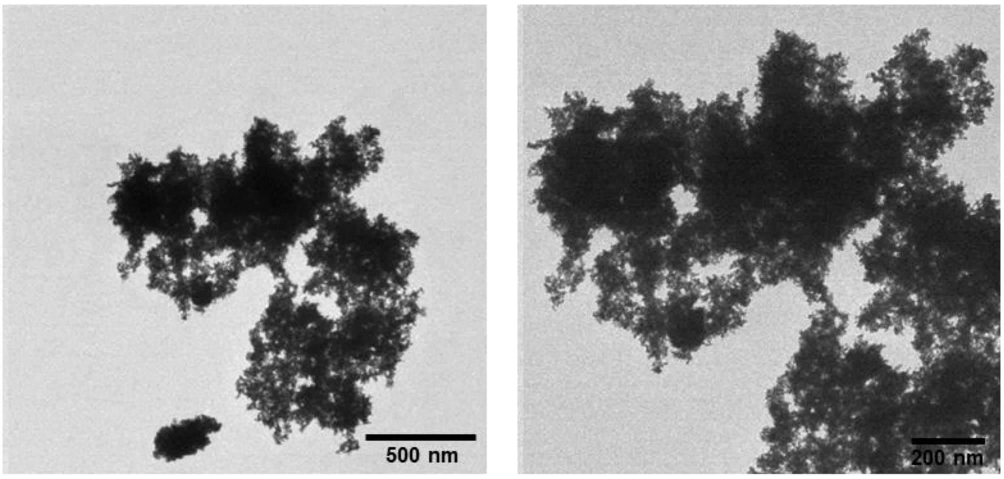

Figure S6 TEM images of Pt black.

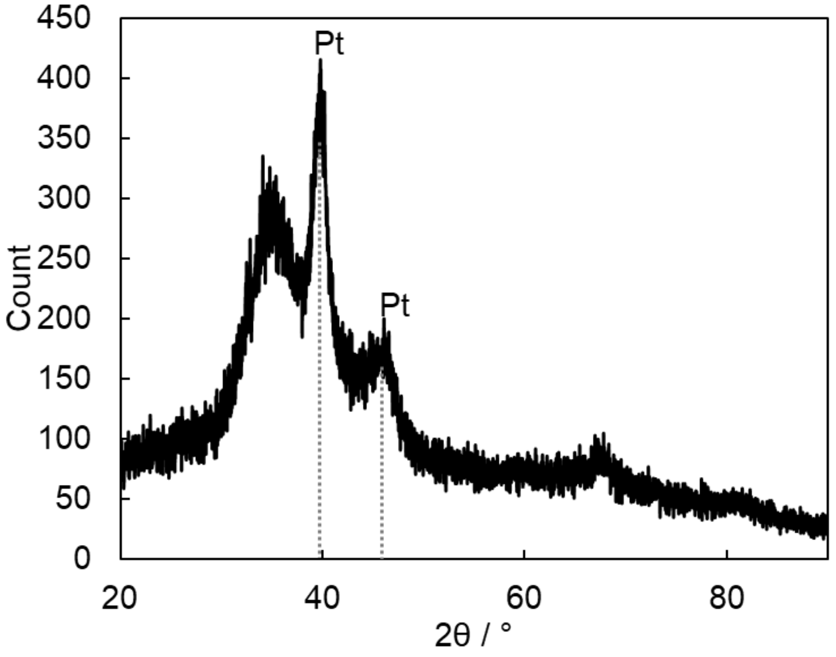

Figure S7 XRD patterns of Pt(dep)-LM obtained by sputtering onto the LM surface for 6 min (1 min x 6 times).

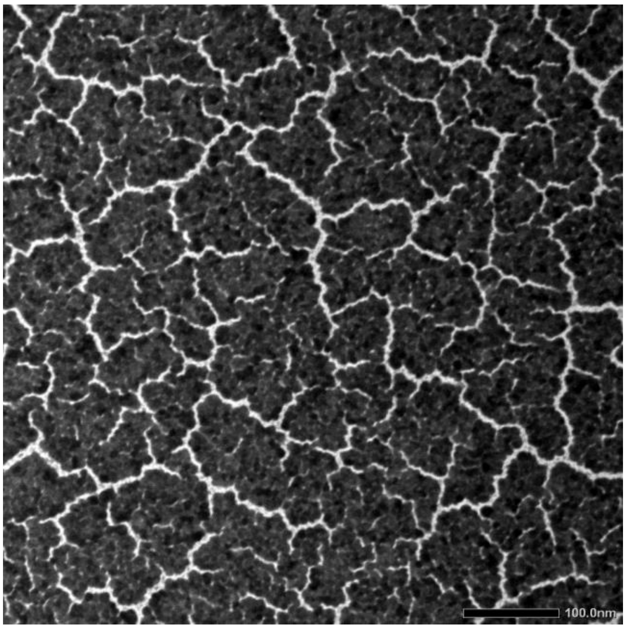

Figure S8 TEM images of Pt obtained by sputtering onto TEM grids for 1 min.

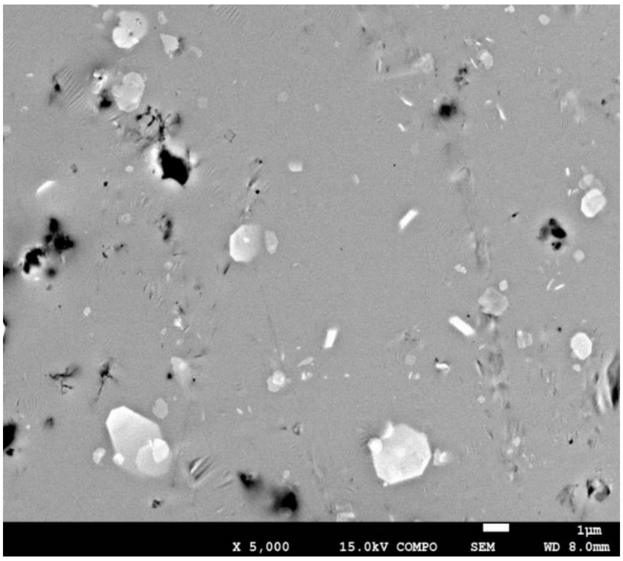

Figure S9 FE-SEM image of Pt(dep)-LM.

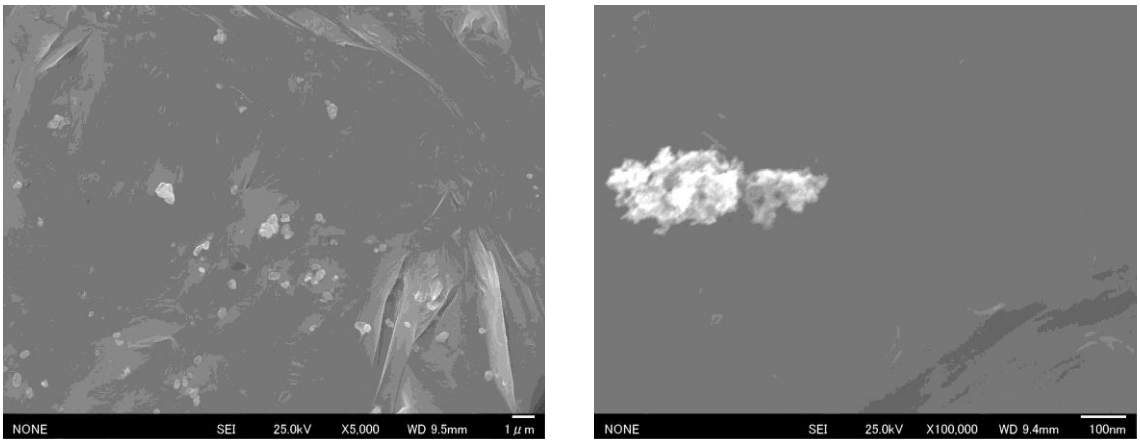

Figure S10 FE-SEM images of Pt(black)-LM.

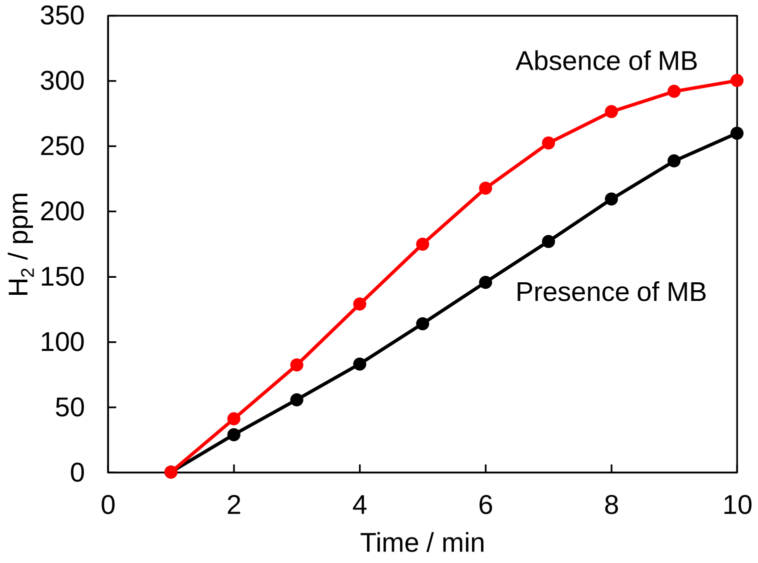

Figure S11 Comparison of the hydrogen production rate of Pt(dep)-LM in the presence and the absence of MB.

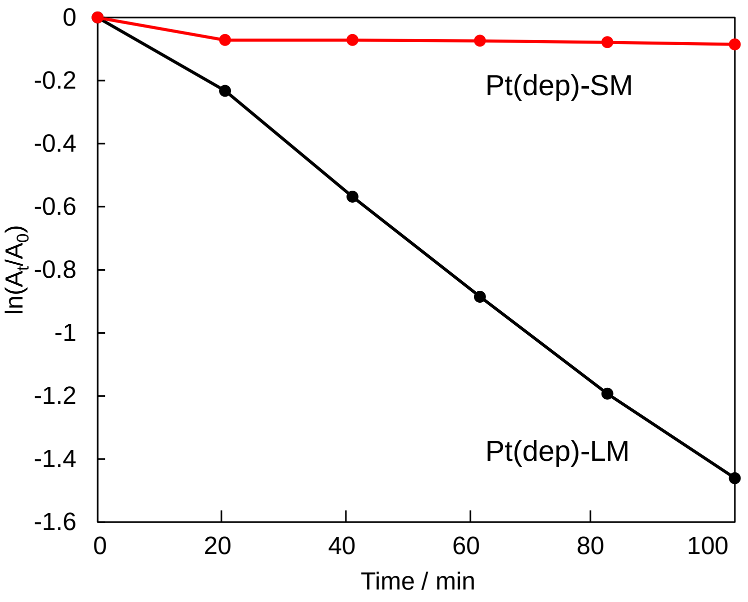

Figure S12 Comparison of MB reduction between Pt(dep)-LM and Pt(dep)-SM.
